# Supplementary material for: Post-earthquake rapid resealing of bedrock flow-paths by concretion-forming resin
Source: Commun Eng. 2024 May 22;3:67. doi: 10.1038/s44172-024-00216-1 (PMC11111762; doi:10.1038/s44172-024-00216-1)
Supplement: Supplementary file 2 — Supplementary information [file 44172_2024_216_MOESM2_ESM.pdf]

Post-earthquake rapid resealing of bedrock flow-paths by concretion-forming resin

Supplementary information

Hidekazu Yoshida[1]*, Koshi Yamamoto[1], Yoshihiro Asahara[2], Ippei Maruyama[2,3], Koichi Karukaya[4], Akane Saito[4], Hiroya Matsui[5], Akihito Mochizuki[5], Mayumi Jo[6], Nagayoshi Katsuta[7], Ayako Umemura[1], Richard Metcalfe[8]

[1]Nagoya University Museum, Nagoya University; Chikusa-ku, Nagoya, Japan

*Corresponding author. Email: dora@num.nagoya-u.ac.jp

[2]Graduate School of Environmental Studies, Nagoya University; Chikusa-ku, Nagoya, Japan
[3]School of Engineering, The University of Tokyo, Tokyo, Japan
[4]Sekisui Chemical Co., LTD; Ritto, Shiga, Japan
[5]Japan Atomic Energy Agency; Horonobe, Hokkaido, Japan
[6]Taisei Corporation; Shinjuku-ku, Tokyo, Japan
[7]Faculty of Education, Gifu University; Gifu, Japan
[8]Quintessa Limited; Newtown Road, Henley-on-Thames, RG9 1HG, Oxfordshire, UK

Supplementary Note 1
Concretion-forming resin "Concretion-seeds"
A liquid epoxy resin (commercial product name: CRJ) is used. The resin contains ions needed to facilitate the formation of a concretion, i.e. it causes calcite precipitation within fractures and pores around the resin-impregnated rock. The concretion-forming resin has been developed under the collaboration work with Sekisui Chemical Co. Ltd building on its accumulated expertise in resin production.

The resin itself increases in hardness a few tens of minutes, and after hardening after mixing the main and hardening agents, the it continuously supplies the source ions for concretion formation until these ions are consumed. The hardening speed and the duration of ion supply for concretion formation (calcite precipitation) after hardening can be controlled by adjusting the resin formulation. For example, the resin can be in the form of liquid and/or pellets, or micron-sized capsules with the size of a few microns to 1 micron (Supplementary Fig. 1). Such micro-capsules type will be effective to inject far distance from the borehole rather than liquid type used. In

particular, the capsule type can use with the mixture of conventional cement-milk type to progress more readily forming calcite around the injected area. Both types can also be varied according the targeted bedrocks flow-paths features. Basically almost all types of bedrocks flow-paths can be sealed by the resin.

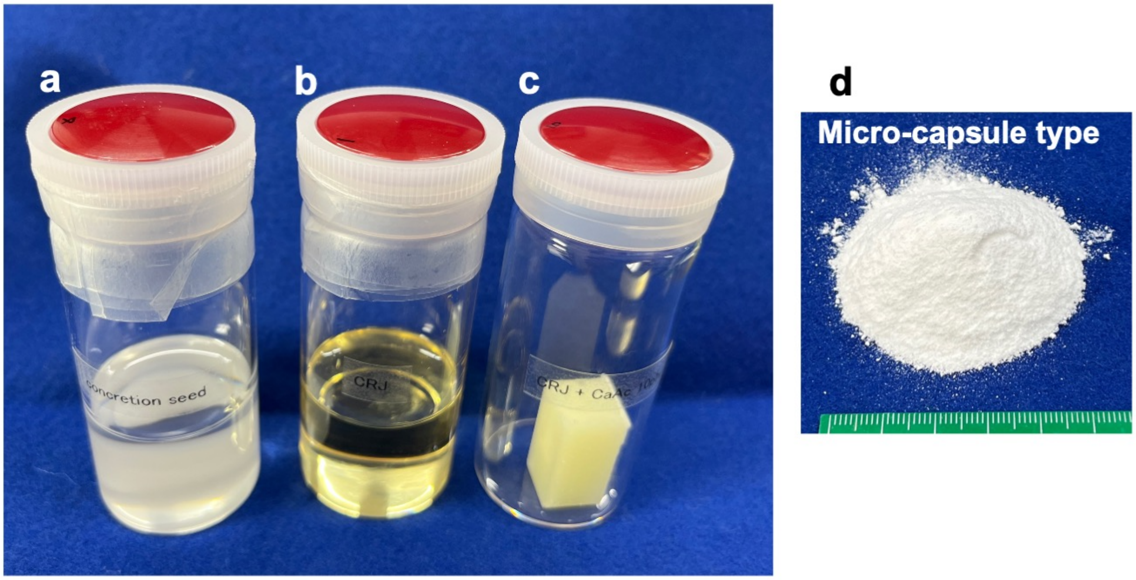

Supplementary Fig. 1: 'Concretion-forming resin' (Concretion-seed; shortened name 'Conseed'; produced in collaboration with Sekisui Chemical Co. Ltd). a,b) Two liquids which are mixed (a: Main Agent; b: Hardening Agent) to produce the concretion-forming resin. c) Hardened concretion-forming resin. d) Micro-capsule type of 'Conseed' can be used with the mixture of conventional grouting materials.

Supplementary Note 2

Layout of *in-situ* experiment, hydraulic test and drilling after experiment

Detailed mapping of fracture distribution around the *in-situ* experimental drift was carried out just after drift excavation. All fractures which have more than 10 cm trace lengths are recorded as shown in Supplementary Figure 2. The location of the *in-situ* hydraulic test was selected based on the fracture distribution. Eight resin injection holes (No.1-8) were drilled with 0.5m diameter surrounding circular hydraulic test center hole. 17 months after resin injection, two resin injected boreholes (No.5 and 6) were over-cored and an additional six boreholes were drilled and cored to confirm the area of sealing caused by the injected ‘concretion-forming resin’ (Supplementary Fig. 3). All boreholes were filled by mortar after over-coring in order to continuously measure the effectiveness of sealing provided by the rest of injected ‘concretion-forming resin’ up to 22 months.

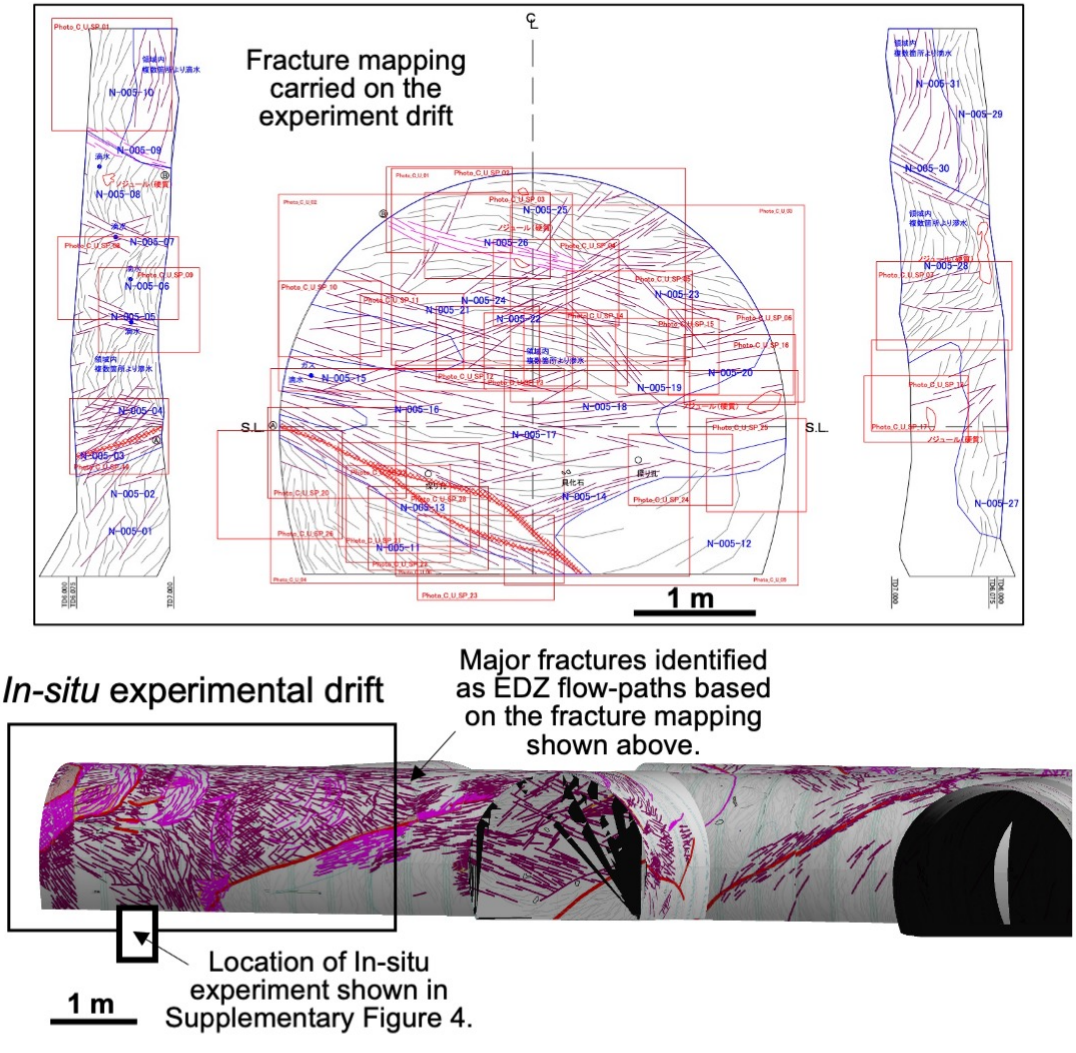

Supplementary Fig. 2: Fracture mapping around the drift excavated at 350 meters depth that was used for the *in-situ* experiment. EDZ: Excavation Damaged Zone.

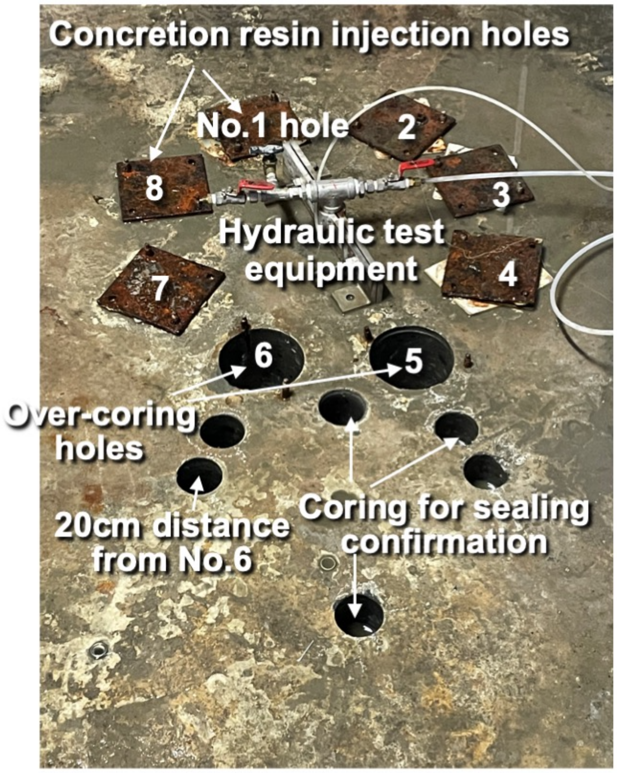

Supplementary Fig. 3: Layout of boreholes and test equipment used in the experimental sealing of the EDZ (Excavation Damaged Zone). Eight resin injection holes (No.1~8) are circular located hydraulic test hole in the center with the diameter of 0.5m. 17 months after resin injection, two of the boreholes (No. 5 and 6) were over-cored and six boreholes were additionally drilled in order to investigate how far calcite sealing has been extended. All those boreholes were filled by mortar immediately after drillings.

Supplementary Note 3

Hydraulic conductivity test of EDZ using water injection

A steady-state hydraulic conductivity test with a packer was located in a borehole that had been drilled through the concrete floor of the tunnel into the EDZ (Supplementary Fig. 4). The results of test are summarized in Supplementary Table 1.

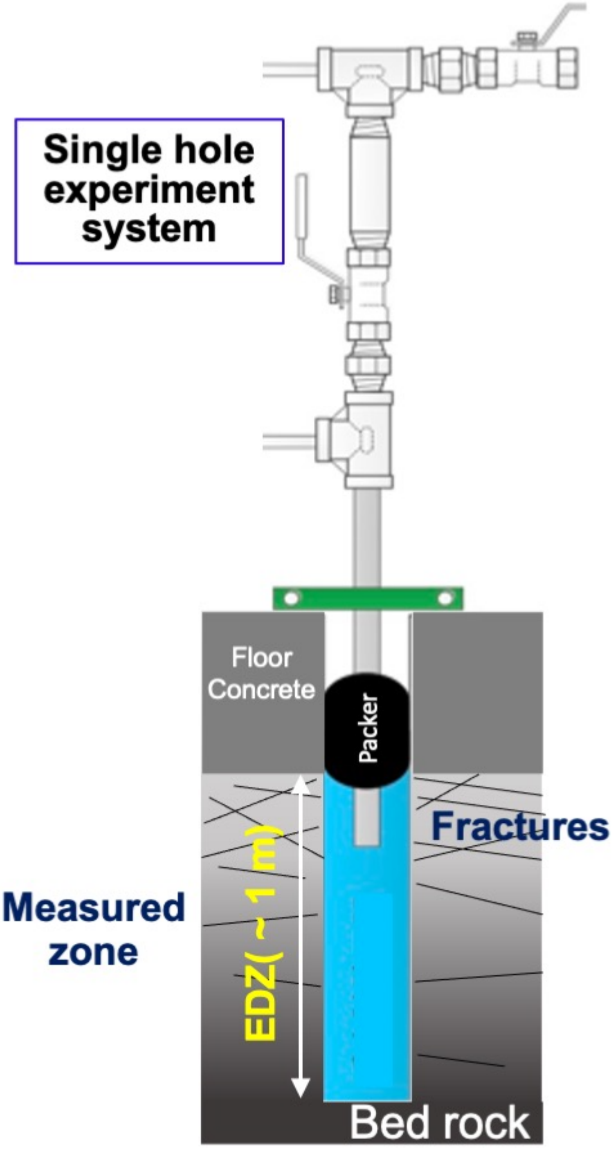

Supplementary Fig. 4: Image of hydraulic test equipment. EDZ: Excavation Damaged Zone.

Date of measurement	Hydraulic conductivity (m/s)
Oct.8th.2021	5.95E-05
Oct.14th.2021	2.35E-06
Jan.26th.2022	4.85E-07
May.27th.2022	3.90E-07
Aug.24th.2022	1.20E-06
Oct.25th.2022	4.01E-07
Nov.15th.2022	2.72E-07
Feb.17th.2023	3.19E-07
Jun.1st. 2023	2.93E-07
Oct.18st. 2023	2.90E-07

Supplementary Table 1:
Hydraulic conductivity measured before and after earthquakes that occurred during the *in-situ* test of concretion-forming resin.

Supplementary Note 4

Earthquake data during the experiment (11th to 12th of August, 2022)

A total of 6 inland earthquakes occurred within two days (11th to 12th of August, 2022). The maximum magnitude was 5.4 (11th Aug. 00:53; at the URL site the magnitude was 5-upper on the Japanese scale of earthquake intensity, which has a maximum magnitude of 7), and the epicenters were at the URL site, with the foci being 2 to 7 km depth below the site. Records of these 6 earthquakes at the URL site are shown in Supplementary Figure 5. Due to these earthquakes, pore-water pressures at the 350 meters level drift increased by up to 75kPa compared to the measured average level before the earthquakes (Supplementary Fig. 6).

During two days, 11 inland earthquakes epicenter at the URL (Underground Research Laboratory) site area and the foci at 2-7 km depth below the site with the maximum Mw 5.4.

Time and days	Depth of epicenter (km)	Magnitude (Mw)	Max earthquake intensity	In-situ
8/11 00:35	2	5.2	5 lower	3
8/11 00:53	5	5.4	5 upper	4
8/11 01:04	6	3.9	3	2
8/11 02:14	6	4.6	4	3
8/11 19:45	7	3.4	2	1
8/12 13:37	2	4.4	3	2

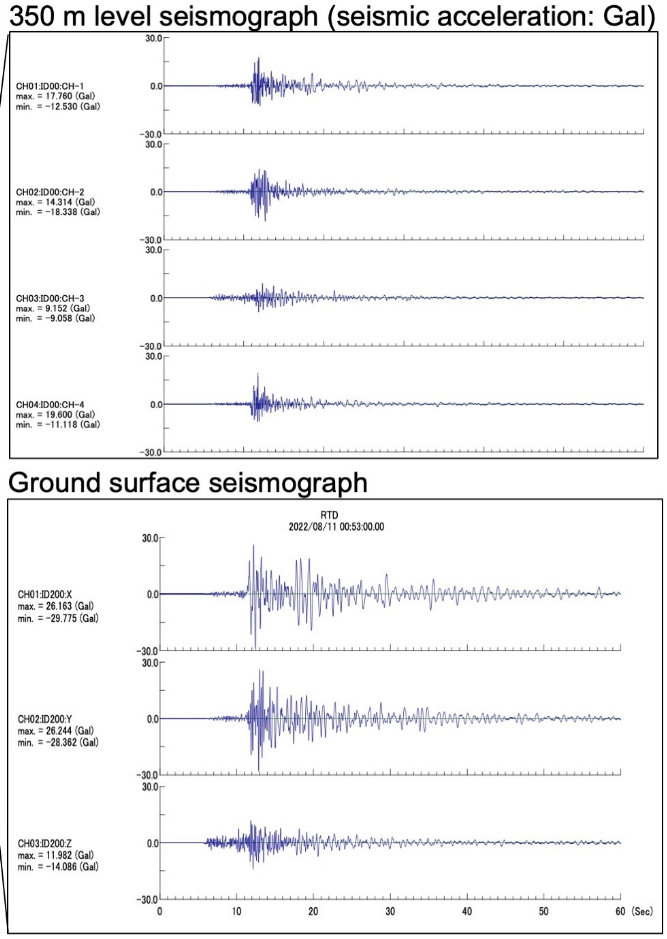

Supplementary Fig. 5: Records of inland earthquake shocks at the Horonobe site between 11th and 12th August 2022. Data source from https://www.jawa.go.jp/04/horonobe/status/cyousa/cyousakenkyu_0408.html.

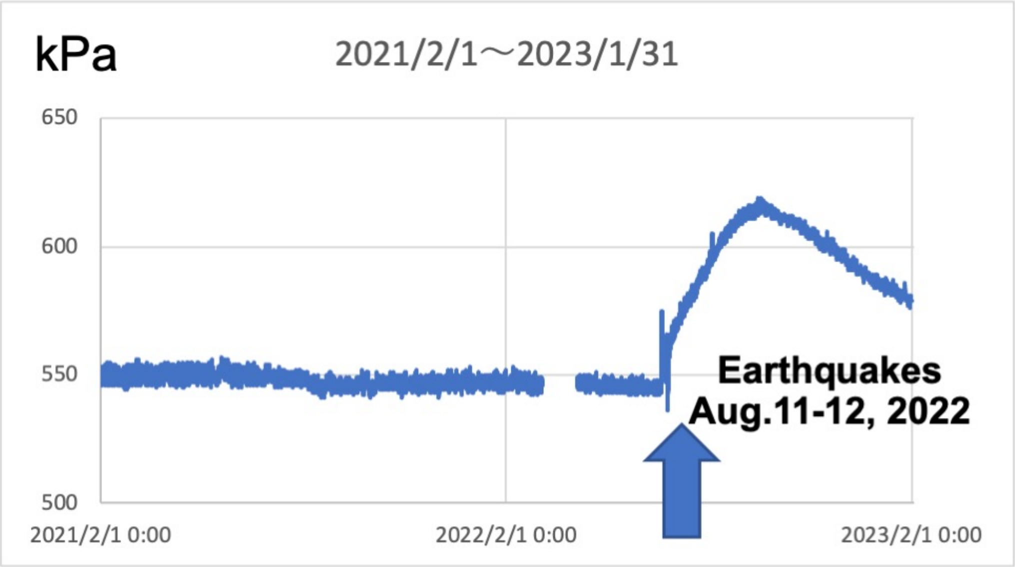

Supplementary Fig. 6: Porewater pressure monitoring in the in-situ test drift at 350 meters depth.

Supplementary Note 5

BTV observation of over-coring holes

BTV observations were carried out in the over-cored boreholes to identify flow-paths sealed by calcite due to injection of the concretion-forming resin (Supplementary Fig. 7). It was observed that almost all fractures were filled by whitish colored calcite. These fractured rocks were sampled and used for XRD analysis and SEM observation.

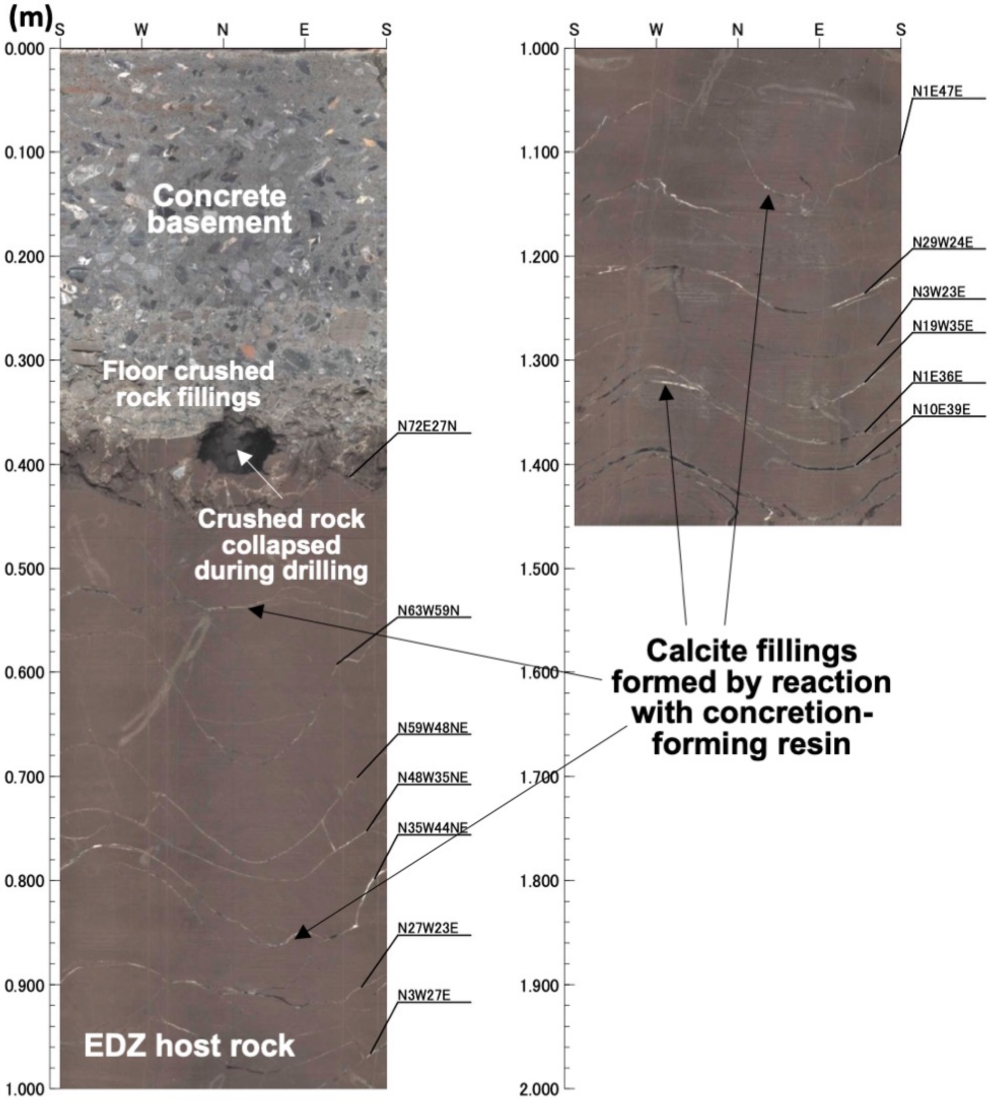

Supplementary Fig. 7: BTV (Bore-hole Television) images of over-cored boreholes into which the 'concretion-forming resin' had been injected. All whitish colored fracture fillings are calcite formed by reaction between the resin and groundwater. EDZ: Excavation Damaged Zone.

Supplementary Note 6

Permeability test by using over-coring core

Permeability of the over-coring core containing resin was carried out by Flow-pump permeability test in Kajima Technical Research Institute (Supplementary Fig. 8). Result shows that the quite low permeability varies from $1.1\text{-}10^{-10}$ m/s to $2.0\text{-}10^{-10}$ m/s almost the same value of permeability measurement carried out on undisturbed core specimens of Wakkanai Formation (40).

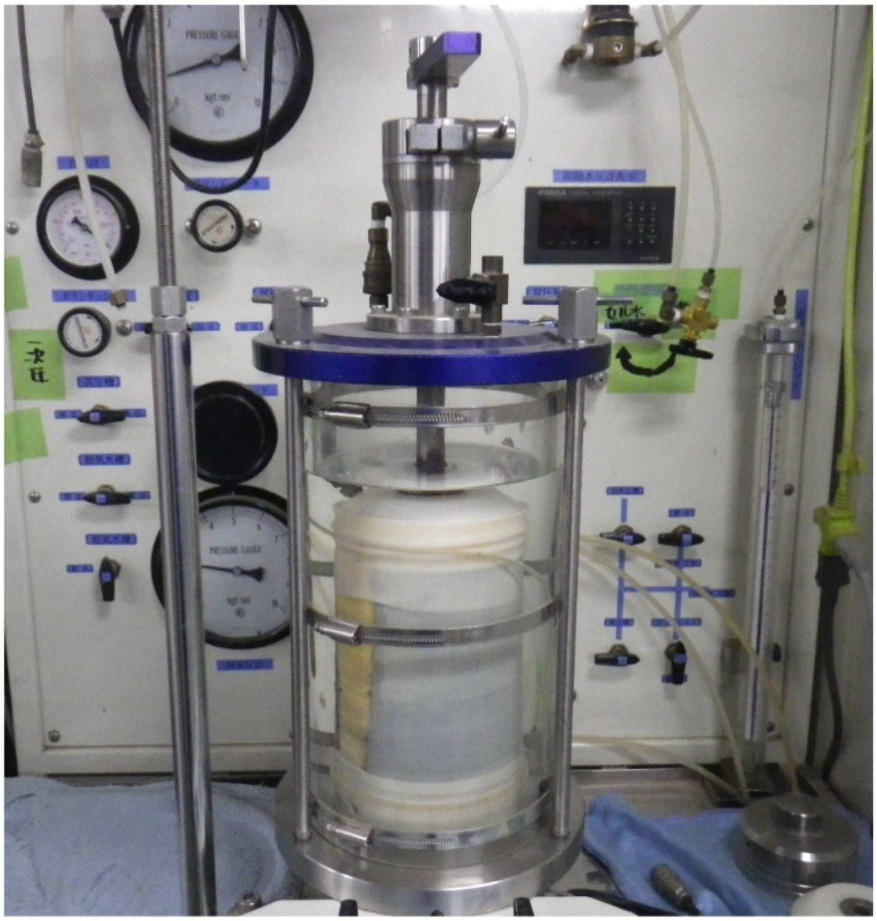

Supplementary Fig. 8: Flow-pump permeability test carried out in Kajima Technical Research Institute with the over-cored core (φ:10cm, length 5cm).

Supplementary Note 7

SEM analysis on synthetically formed calcite fillings in flow-paths

SEM observations of fractures filled with calcite were carried out to determine the physical characteristics of the calcite. Almost all fractures were filled by euhedral calcite crystals growing from the fracture walls (Supplementary Fig. 9).

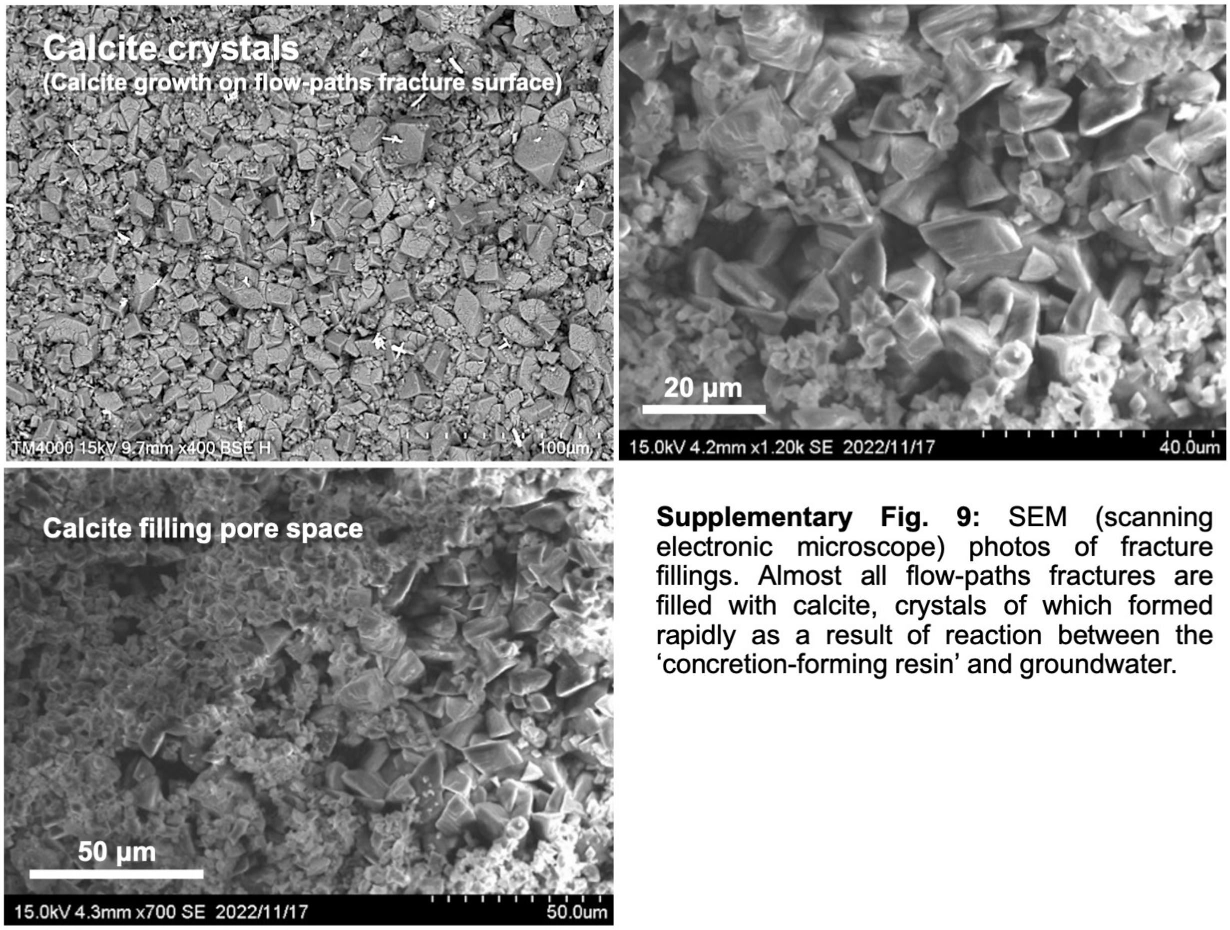

Supplementary Fig. 9: SEM (scanning electronic microscope) photos of fracture fillings. Almost all flow-paths fractures are filled with calcite, crystals of which formed rapidly as a result of reaction between the 'concretion-forming resin' and groundwater.

Supplementary Note 8
X-ray diffraction (XRD)
The mineralogical compositions of the fracture fillings were determined with an X-ray diffractometer (XRD; Multiflex, Rigaku Co.) using crushed and powdered samples and Cu Kα radiation, an electron beam of 40 kV/20 mA) (Supplementary Fig. 10).

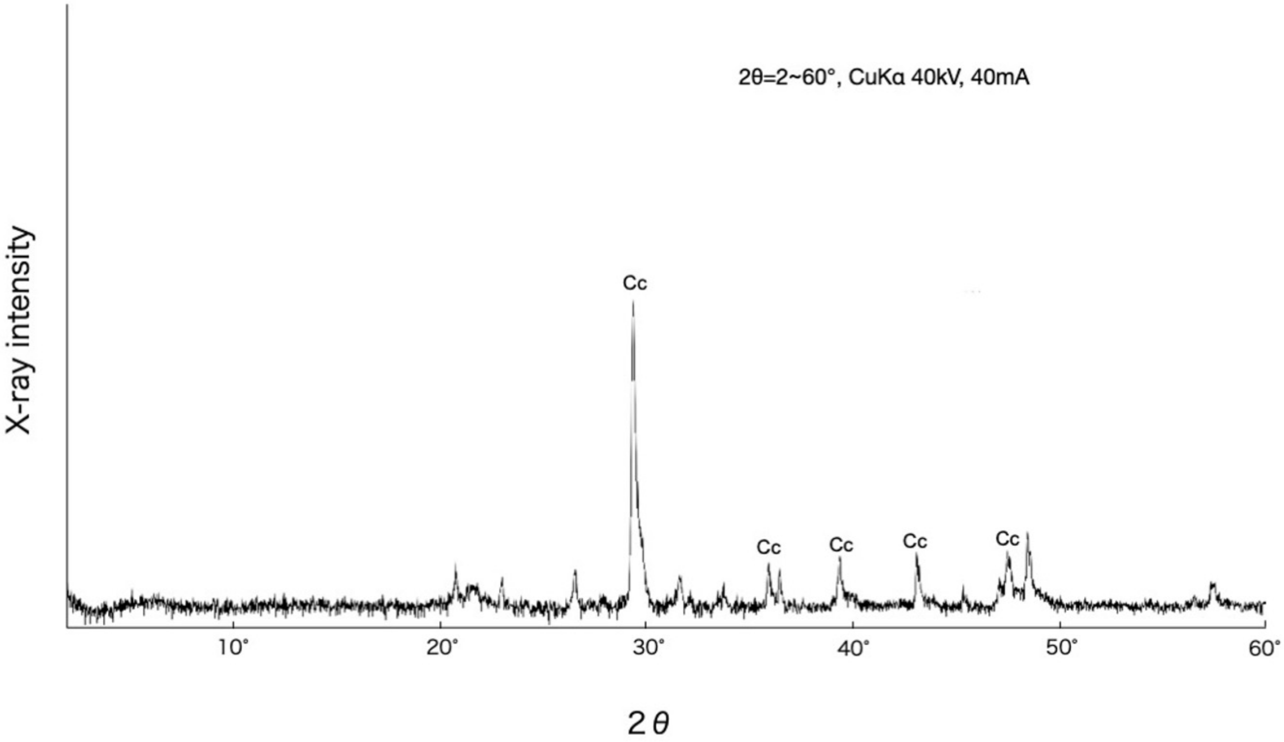

Supplementary Fig. 10: X-ray diffractograms given by fracture fillings (Cc: Calcite).

Supplementary Note 9

X-ray analytical microscopy (SXAM)

Cross-sections of over-cored core were used to determine elemental concentration profiles across the concretion-forming resin and surrounding rock matrices using an X-ray fluorescence analyzer (SXAM: XGT-5000V Horiba Japan). The results showed semi-quantitatively the two-dimensional distribution of Si, Al, Fe, Mn and K across the whole surface of each sample (Supplementary Fig. 11).

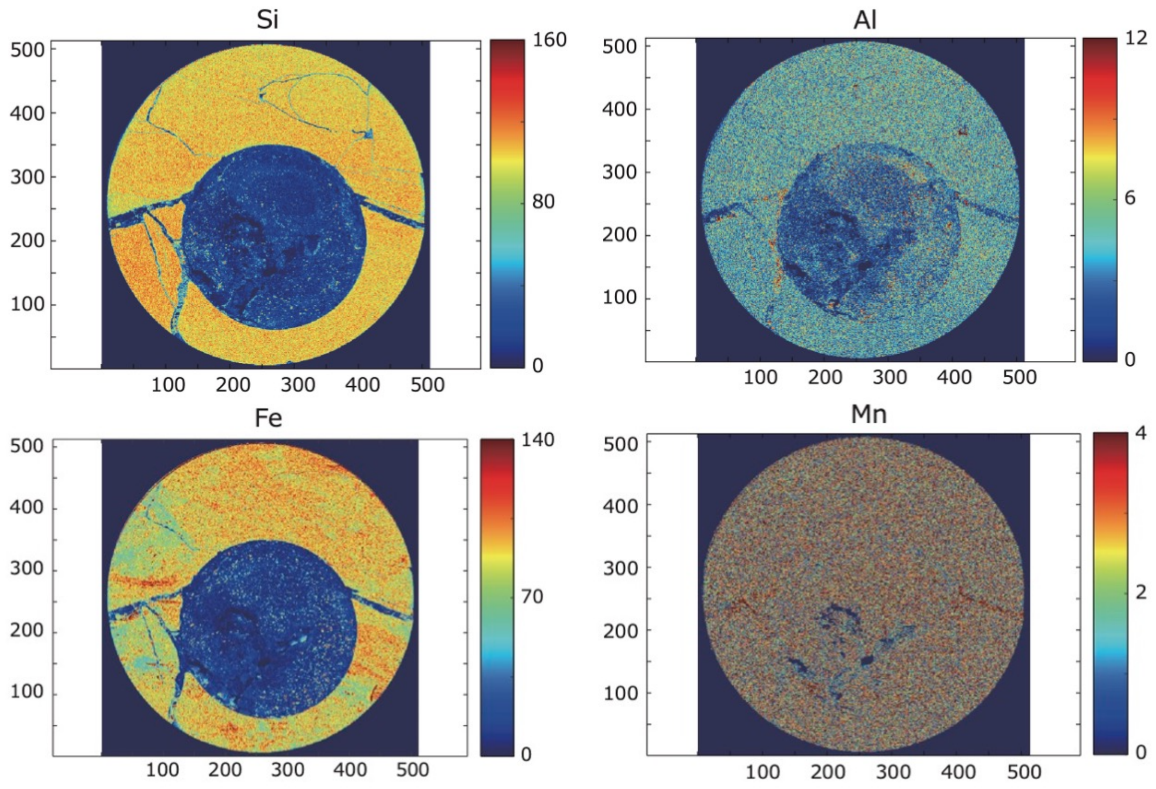

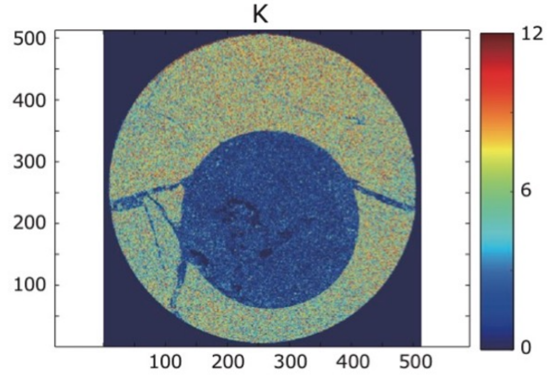

Supplementary Fig. 11: SXAM elemental mapping of Si, Al, Fe, Mn and K in the rock cores obtained by over-coring after the experiment had been running for 17 months. The colored scale shows the X-ray intensity. Ca mapping is shown in Figure 5 in the text.
Core diameter = 10cm.

Supplementary Note 10

Ca concentration measurement of resin before and after the experiment

<Laboratory experiment>

The rate of Ca leaching from 'concretion-forming resin' has been measured during 3 months by free-water laboratory leaching experiment. Measurement has been carried out by following process. The 8.0 g of concretion-forming resin containing 50 phr of $CaCl_2$ was placed in 250 ml of ultrapure water to determine the rate of Ca elution. Concentrations of Ca^{2+} ion in the water were determined by the ICP-MS. After 3 months, about 36% of Ca^{2+} ion was leached from the resin (Supplementary Fig. 12).

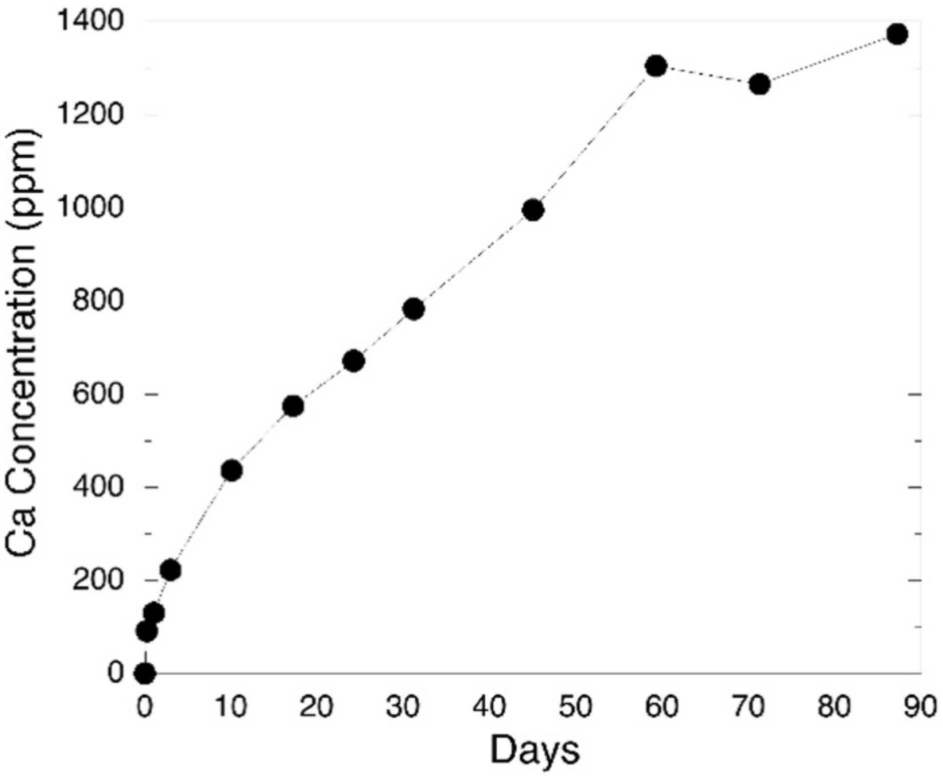

Supplementary Fig. 12: Leaching rate of Ca from 'Concretion-forming resin' during three months.

<*In-situ* experiment>

The Ca concentration of the over-cored concretion-forming resin was also measured to confirm the quantity of Ca consumed during the experiment. The resin was combusted at 350℃ and Ca was dissolved in 2 %-HNO_3 for analysis. The purpose was to estimate the period for which the sealing process would continue. Result shows that only ca. 20% of Ca^{2+} ion was consumed and suggests that the sealing effect will be continued at least for 4-5 times duration than monitoring period (Supplementary Table 2).

	Ca content (wt%)
Original resin	7.25
After experiment (over-cored core)	6.00 center<br>5.89 inner middle<br>5.56 outer middle<br>5.47 outer rim

Supplementary Table 2: Ca-content of the ‘concretion-forming resin’ before and after the *in-situ* experiment.

Supplementary Note 11

Synthetic calcite sealing examined by laboratory experiment

Experiment of ‘Concretion-forming resin’ with glass beads and distilled water was carried out in order to examine the calcite formation and sealing properties of pores among glass beads. The result shows that the calcite precipitation is rapidly progressed as shown in Supplementary Figures 13a-d. Within a week, calcite crystals forms on glass beads and continuously grown and cemented the pores during three months.

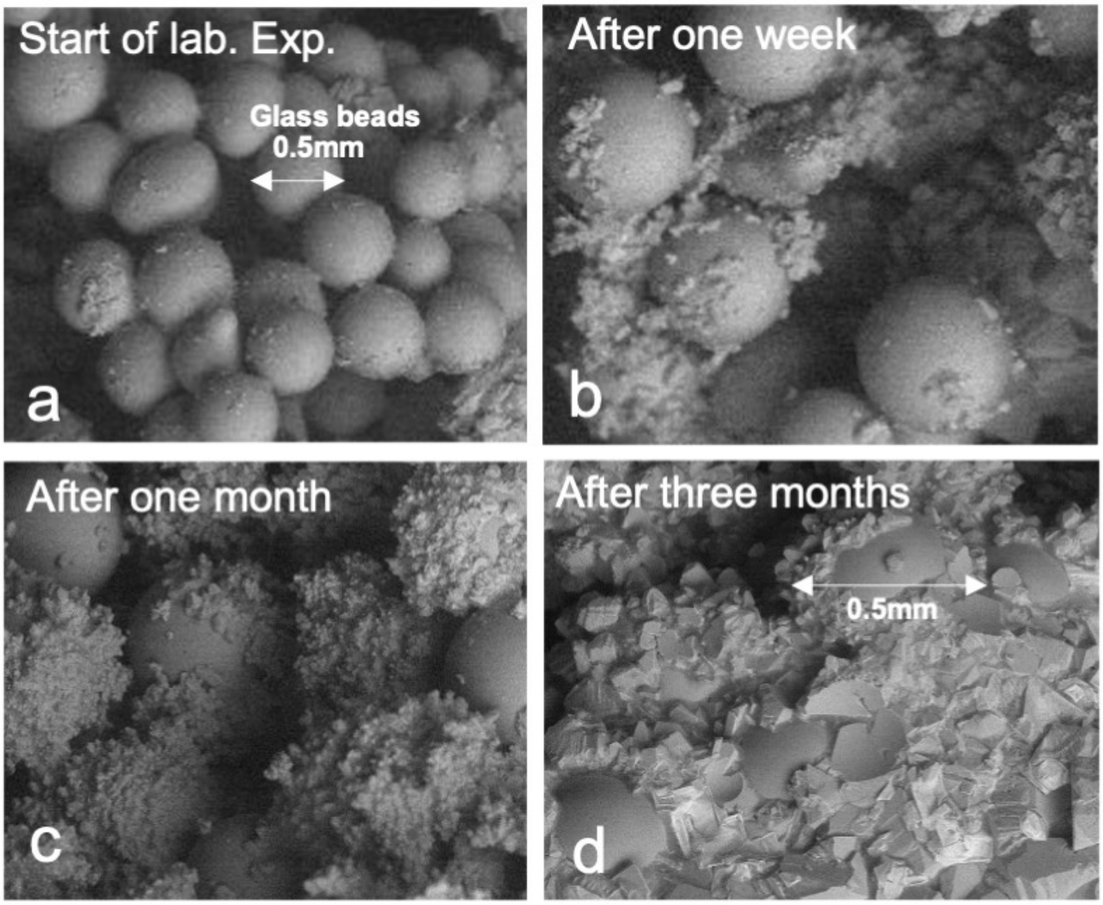

Supplementary Fig. 13: SEM (scanning electronic microscope) photos of calcite cementation developed rapidly by super-saturation with HCO_3^- and Ca^{2+} supplied from the ’concretion-forming resin’ and groundwater.
